# PICH deficiency attenuates the progression of lung adenocarcinoma and disrupts the DNA damage response

**DOI:** 10.1002/ctm2.70349

**Published:** 2025-05-29

**Authors:** Li Miao, Lu Weina, Lou Jiafei, Chen Gaoying, Yu Yinghui, Wu Yinfang, Li Fei, Zhang Chao, Tang Haoyu, Weng Qingyu, Zheng Kua, Gao Shenwei, Wu Yanping, Zhu Chen, Zhang Min, Yu Fangyi, Jin Rui, Chen Zhihua, Geng Xinwei, Ying Songmin, Li Wen

**Affiliations:** ^1^ Key Laboratory of Respiratory Disease of Zhejiang Province, Department of Respiratory and Critical Care Medicine Second Affiliated Hospital of Zhejiang University School of Medicine Hangzhou Zhejiang China; ^2^ Surgical Intensive Care Unit Second Affiliated Hospital of Zhejiang University School of Medicine Hangzhou Zhejiang China; ^3^ Department of Pharmacy Center for Regeneration and Aging Medicine, the Fourth Affiliated Hospital of School of Medicine, and International School of Medicine, International Institutes of Medicine Yiwu China; ^4^ Zhejiang‐Denmark Joint Laboratory of Regeneration and Aging Medicine Yiwu Zhejiang China

1

Dear Editor,

In this study, we showed that the DNA helicase PICH is essential for the progression of *Kras^G12D^*‐driven lung adenocarcinoma in vivo and for the growth of human lung adenocarcinoma cells in vitro. These findings suggest that PICH might be a promising therapeutic target in lung adenocarcinoma.

Chromosomal instability is widely recognised as a hallmark of cancer.^1^ Disrupting pathways that regulate chromosomal stability offers a potential strategy for cancer therapy. Lung cancer is the leading cause of cancer‐related death worldwide.^2^ Lung cancer patients, particularly those with advanced disease, still face a poor prognosis and a dearth of effective treatment strategies. PICH, a member of the SNF2 family of ATPases, is critical for maintaining chromosomal stability by facilitating mitotic chromosome organisation and segregation.^3^ Recently, several studies support the notion that PICH is essential for the proliferation of certain cancer cell types and is associated with unfavourable prognoses in cancer patients.^4^, ^5^ However, the precise role of PICH in lung cancer remains largely undefined due to the limited availability of compelling preclinical evidence, particularly from in vivo primary tumour models. To bridge this gap, we systematically examined the role of PICH in lung cancer through clinical analysis, in vitro experiments, and in vivo primary tumour model to thoroughly examine PICH's involvement in lung cancer and evaluate its potential for therapeutic intervention.

To investigate the expression pattern of PICH in lung cancer, we first analysed publicly available datasets, focusing specifically on the two most common subtypes: lung adenocarcinoma (LUAD) and lung squamous cell carcinoma (LUSC).^6^, ^7^, ^8^ Elevated expression of PICH at both the mRNA and protein levels was observed in LUAD and LUSC tissues (Figures 1A–C and S1A–C). To validate these results, immunohistochemical analysis was performed on 25 paired tumour and adjacent normal lung tissues from LUAD patients, revealing a notable upregulation of PICH in tumour tissues (Figure 1D and E). Additionally, higher PICH expression levels were associated with advanced tumour stages (Figure S2). Next, the prognostic value of PICH in lung cancer patients was evaluated. PICH expression was higher in LUAD patients who died within 3 years of diagnosis compared to those who survived (Figure 1F). In line with these findings, analysis of publicly available datasets revealed that high levels of PICH predicted worse clinical outcomes across multiple survival indicators in LUAD—including overall survival (OS), disease‐specific survival (DSS), disease‐free interval (DFI), progression‐free interval (PFI), first progression (FP), and relapse‐free survival (RFS)—but not in LUSC (Figures 1G–L and S1D–I).

**FIGURE 1:**
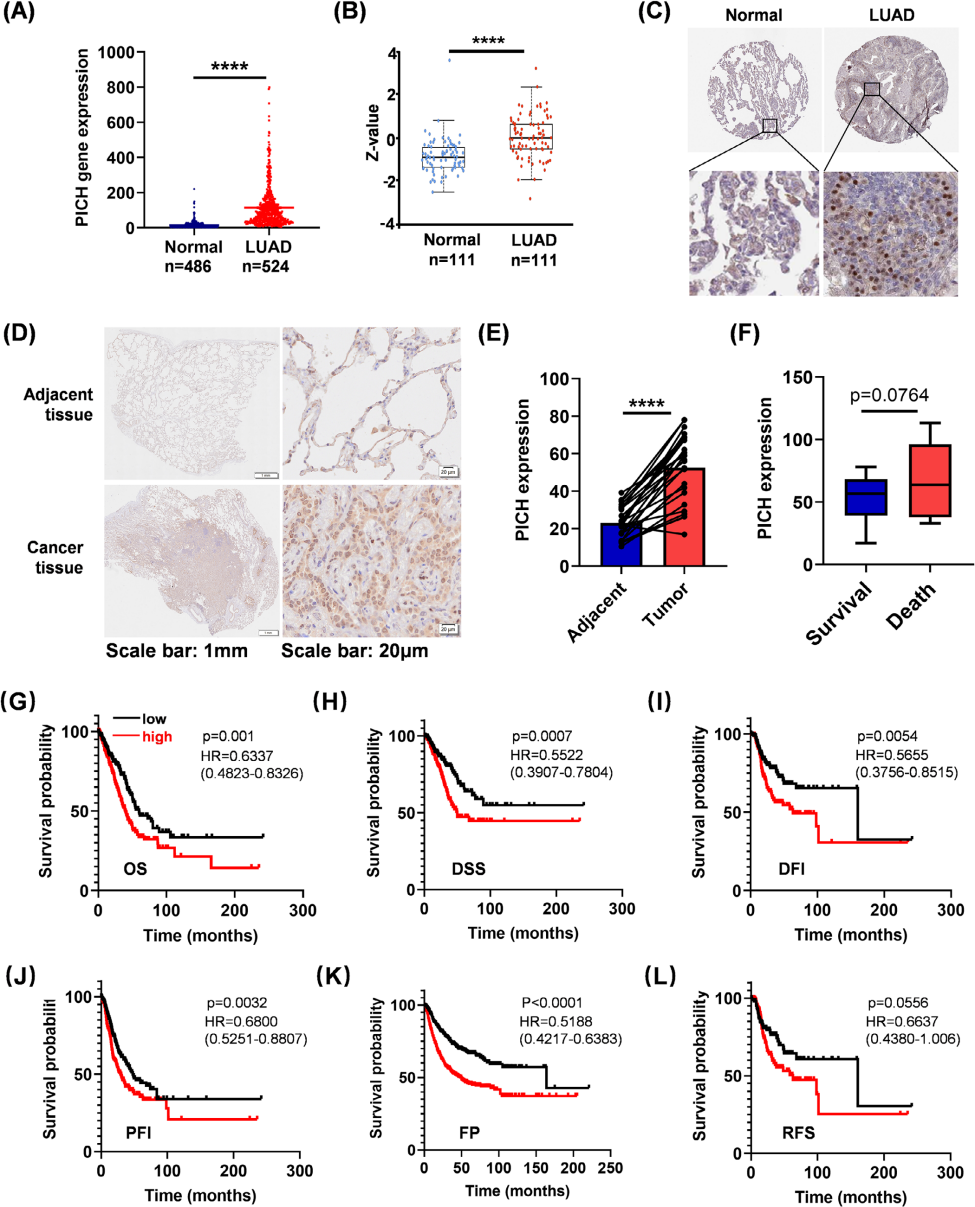
PICH is highly expressed in lung adenocarcinoma patients and indicates poor outcomes. （A）Comparative analysis of PICH mRNA expression in normal lung tissues versus LUAD samples using the Kaplan–Meier Plotter database (https://kmplot.com/). (B) PICH protein levels in normal lung and LUAD tissues were compared using the CPTAC database. (C) Representative immunohistochemical images of PICH expression in normal lung and LUAD tissues sourced from The Human Protein Atlas (https://www.proteinatlas.org/). (D) Immunohistochemical staining of PICH in paired tumour and adjacent non‐tumorous lung tissues derived from patient surgical specimens. (E) Quantification of PICH expression in tumour and adjacent non‐tumorous lung tissues assessed by immunohistochemical staining (adjacent: *n* = 25, tumour: *n* = 25). (F) PICH expression levels in lung adenocarcinoma patients who died within 3 years (*n* = 7) of diagnosis compared to those who survived beyond 3 years (*n* = 23). (G) Overall survival analysis in patients with LUAD was performed by stratifying individuals based on high or low PICH expression. (high: *n* = 281; low: *n* = 282). (H) Disease‐specific survival analysis in patients with LUAD (high: *n* = 262; low: *n* = 266). (I) Disease free interval analysis in patients with LUAD (high: *n* = 151; low: *n* = 187). (J) Progression free interval analysis in patients with LUAD (high: *n* = 282; low: *n* = 283). (K) First progression analysis in patients with LUAD (high: *n* = 432; low: *n* = 442). (L) Relapse free survival analysis in patients with LUAD (high: *n* = 149; low: *n* = 150). Student's *t*‐test analysis was used in A, B, E, F plots, and log‐rank test analysis was used in G‐L plots, ****p < .0001.

A more detailed subgroup analysis demonstrated that elevated PICH expression was significantly linked to decreased overall survival (OS) in LUAD patients with distant lymph node metastasis (N2 vs. N0/1) (Figure S3A). Interestingly, the prognostic impact of PICH overexpression was particularly pronounced in individuals with low neoantigen load, low mutation burden, or those who were non‐smokers (Figure S3B–D). Taken together, these results indicate that PICH overexpression is associated with unfavourable clinical outcomes in LUAD, especially among those with N2 lymph node metastasis, lower neoantigen load, low mutation burden, or non‐smoking status.

To investigate the functional role of PICH in LUAD, we performed loss‐of‐function experiments in A549 and H1299 lung adenocarcinoma cells using a lentivirus‐mediated shRNA delivery system (Figure 2A). The deletion efficiency of PICH was validated using western blot analysis (Figure 2B). Functional assays revealed that PICH downregulation significantly impaired the proliferation and clonogenic capacity of lung adenocarcinoma cells (Figure 2C–E). Moreover, PICH deficiency was found to induce apoptosis in lung adenocarcinoma cells (Figure 2F–G). These findings underscore the indispensable role of PICH in the proliferation and survival of lung adenocarcinoma cells.

**FIGURE 2:**
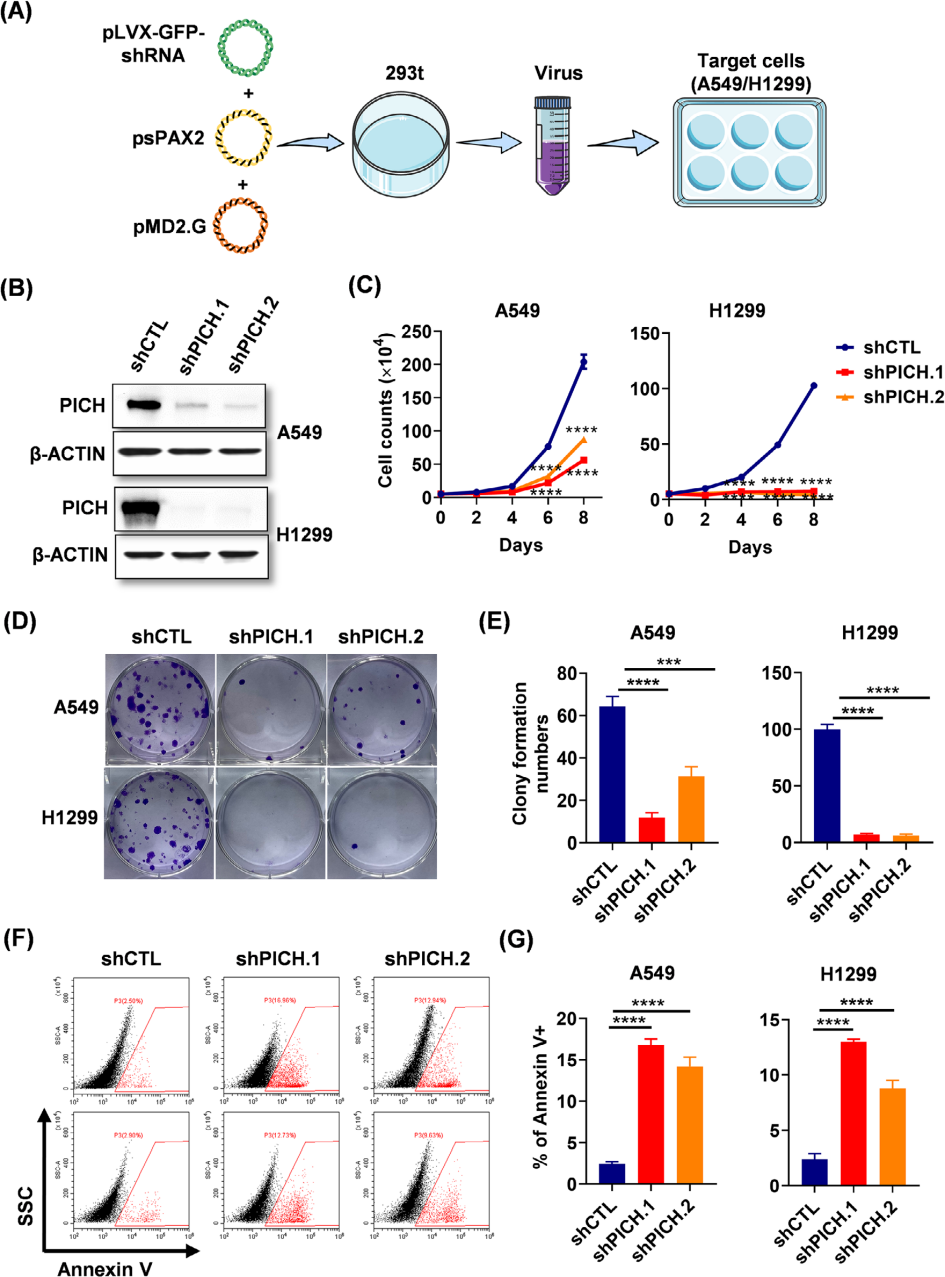
PICH depletion abrogates the proliferation and survival of lung adenocarcinoma cells. （A）Construction of PICH stable knockdown cell lines (Image provided by Servier Medical Art (https://smart.servier.com/), licensed under CC BY 4.0 (https://creativecommons.org/licenses/by/4.0/)). Two PICH shRNAs were ligated to the pLVX‐ZsGreen‐GFP plasmid and transfected into 293t cells using the lentiviral packing plasmids psPAX2 and pMD2.G. After 48 h transfection, the cellular supernatant was collected and used to infect A549/H1299 cells. (B) Western blotting of whole‐cell extracts from control or PICH knockdown lung adenocarcinoma cells; β‐ACTIN was used as a loading control. (C) Proliferation curve of PICH knockdown lung adenocarcinoma cells. (D) Colony formation assay of lung adenocarcinoma cells with stable PICH knockdown. (E) Statistical diagram of the colony formation assay. (F) Apoptosis analysis of PICH knockdown lung adenocarcinoma cells by flow cytometry. (G) Statistical diagrams of apoptosis analysis. The data were presented as the mean ± SEM. One‐way ANOVA was used in C, E, and G plots. ****p < .0001, ***p <.001.

PICH is known to maintain chromosomal stability by modulating the structure and segregation of mitotic chromosomes. To assess the consequences of PICH deficiency in lung adenocarcinoma cells, DNA damage in PICH‐knockdown cells was first assessed using the comet assay. The results revealed a significant increase in DNA strand breaks in PICH deficient cells (Figure 3A and B). Consistently, the DNA damage response marker γH2AX was markedly elevated in the absence of PICH (Figure 3C and D). More importantly, micronuclei—indicative of chromosomal instability—were significantly elevated in PICH‐depleted cells. (Figure 3C and E). Given that DNA damage can induce apoptosis through both p53‐dependent and independent mechanisms,^9^ apoptosis‐related proteins in PICH‐silenced cells were sequentially examined. In p53 wild‐type A549 cells, levels of P53, PUMA, and cleaved caspase‐3/7/9 were notably elevated. In p53‐deficient H1299 cells, activation of PARP and caspases were also observed (Figure 3F). These findings suggest that in lung adenocarcinoma cells, PICH loss induces apoptosis through mechanisms that may involve both p53‐dependent and independent pathways. Overall, the accumulation of DNA damage and chromosome instability resulting from PICH depletion likely contributed to reduced cell proliferation and increased apoptosis in lung adenocarcinoma cells.

**FIGURE 3:**
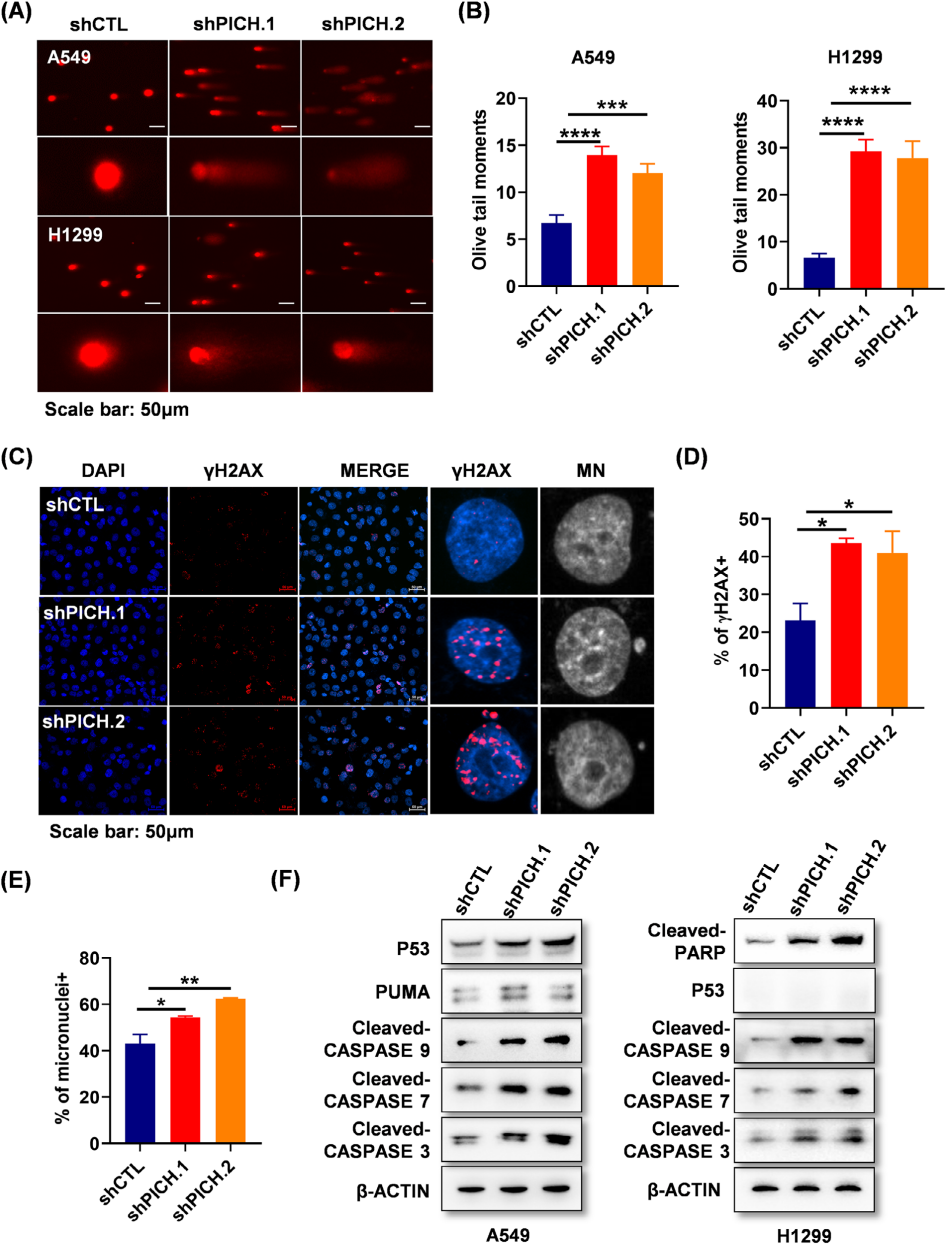
Loss of PICH triggers aberrant DNA damage and chromosome instability in lung adenocarcinoma cells. (A) Representative comet assay images illustrating DNA damage in A549 and H1299 cells following PICH knockdown. (B) Quantification of DNA damage levels based on comet tail measurements. (C) Immunofluorescent staining of γH2AX and micronuclei (MN) in H1299 cells with PICH silencing. (D) Proportion of γH2AX‐positive cells. (E) Proportion of micronuclei‐positive cells. (F) Expression profiles of apoptosis‐related proteins in cells following PICH knockdown. Data are presented as mean ± SEM. Statistical analysis in panels B, D, and E was performed using one‐way ANOVA. ****p <.0001, ***p <.001, **p <.01, *p < .05.

KRAS mutations are well‐established drivers of lung adenocarcinoma. Among various models, *Kras^G12D^* mice are widely used in lung adenocarcinoma research due to their ability to spontaneously develop lung tumours that closely mimic human disease in both progression and morphology.^10^ To assess the function of PICH in lung tumourigenesis in vivo, *Pich^flox/flox^* mice were crossed with *Kras^G12D^* mice to generate *Kras^G12D^*‐*Pich^flox/flox^* mice, with *Kras^G12D^* mice serving as the control group (Figure 4A). The oncogenic *Kras^G12D^* allele was activated via airway instillation of Ad‐Cre virus (Figure 4B). Upon establishment of the lung adenocarcinoma model, PICH expression was assessed in lung tumours from both *Kras^G12D^* and *Kras^G12D^*‐*Pich^flox/flox^* mice. The results showed that PICH was successfully ablated in tumours from *Kras^G12D^*‐*Pich^flox/flox^* mice, while its expression was markedly upregulated in tumours from *Kras^G12D^* mice (Figure 4C), consistent with the observations in human lung cancer samples. Notably, PICH deletion significantly reduced tumour burden and infiltration (Figure 4D and E), suggesting that PICH promotes lung adenocarcinoma progression. Furthermore, PICH deficiency led to a marked reduction in Ki67‐positive cells, along with elevated levels of   γH2AX and cleaved caspase‐3, indicating elevated DNA damage and apoptosis (Figure 4F and G). These results are consistent with our in vitro findings, reinforcing the notion that PICH contributes to tumour cell viability by limiting excessive DNA damage. Remarkably, PICH knockout mice exhibited a significantly extended lifespan compared to PICH wild‐type mice (Figure 4H), highlighting the potential therapeutic value of targeting PICH for the treatment of lung adenocarcinoma.

**FIGURE 4:**
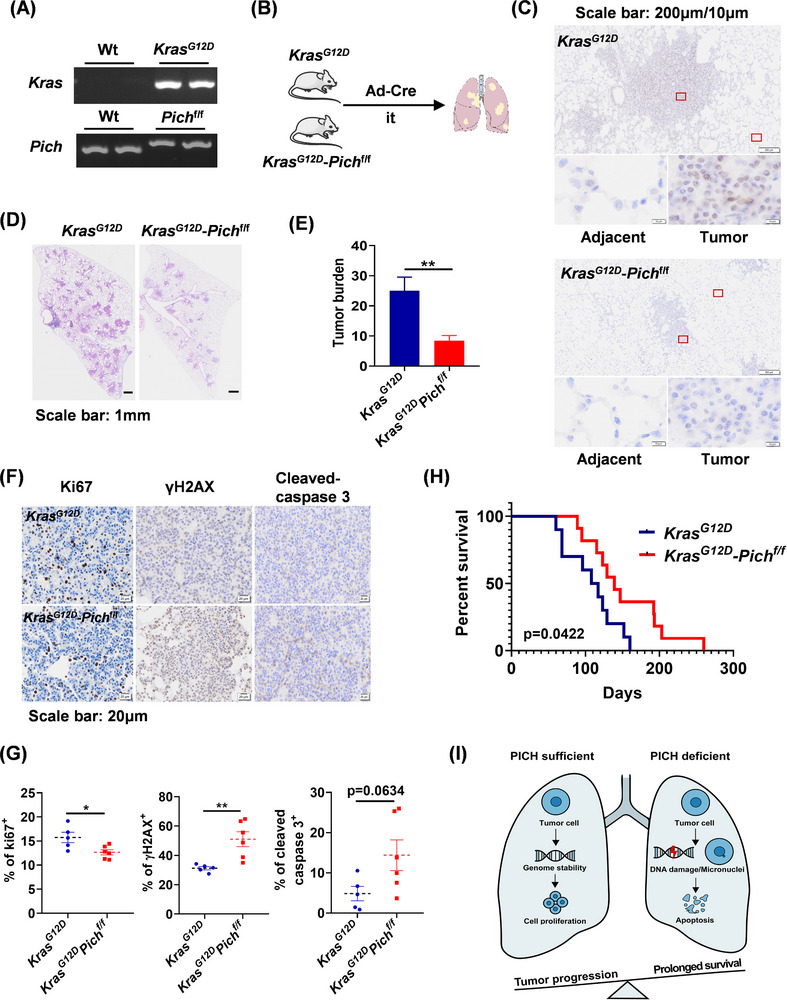
PICH promotes the progression of lung adenocarcinoma in transgenic mouse model. (A) Genotyping images of *Kras^G12D^* and *Pich^flox/flox^* mice. (B) The process of lung adenocarcinoma mouse model construction. Ad‐Cre was intratracheal (it) instilled into 6‐ to 8‐week‐old mice. (C) Representative immunohistochemistry staining for PICH in lung tissues from *Kras^G12D^* and *Kras^G12D^‐Pich^flox/flox^* mice following intratracheal administration of Ad‐Cre. (D) Representative picture of lung tissues stained with Hematoxylin‐Eosin. (E) Statistics of tumour burden, tumour area/lung area (𝑛 = 7 per group); (F) Immunohistochemistry staining for Ki67, γH2AX, and cleaved caspase 3 in lung tumours. (G) Quantification of the percentage of Ki67, γH2AX and cleaved caspase 3‐positive cells (*n* = 5 and *n* = 6, respectively). (H) Survival curves of *Kras^G12D^* (*n* = 10) and *Kras^G12D^‐Pich^flox/flox^* (*n* = 11) mice instilled with Ad‐Cre. (I) Schematic diagram of current study. PICH deficiency in lung adenocarcinoma cells leads to increased DNA damage and chromosomal instability, which activates apoptotic pathways and consequently inhibits tumour progression. The data were presented as means ± SEM. Student's *t*‐test was used in E and G plots, log‐rank test was used in H plot. **p < .01, *p < .05.

In conclusion, we first demonstrated the pivotal role of PICH in lung adenocarcinoma by establishing a primary tumour model using PICH conditional knockout mice. Our results demonstrate that PICH is overexpressed in lung adenocarcinoma and correlates with poor patient prognosis. We further show that PICH promoted the proliferation and survival of lung adenocarcinoma cells by preventing excessive DNA damage and chromosomal instability. Notably, PICH deficiency markedly suppressed tumour progression and prolonged survival in mice, providing innovative insights into the potential therapeutic targeting of PICH for lung adenocarcinoma. Our study provides valuable mechanistic understanding of lung adenocarcinoma pathogenesis and reinforces the rationale for targeting chromosomal stability regulators as a promising therapeutic strategy.

## AUTHOR CONTRIBUTIONS

Wen Li, Songmin Ying, Xinwei Geng, and Zhihua Chen conceived and designed this study. Miao Li, Weina Lu, and Jiafei Lou contributed equally to this work. Miao Li, Jiafei Lou, Gaoying Chen, and Yinghui Yu carried out the in vitro experiments. Miao Li, Qingyu Weng, Kua Zheng, Shenwei Gao, and Fangyi Yu performed the in vivo experiments. Weina Lu, Yinfang Wu, Fei Li, and Chao Zhang assisted with the statistical analyses. Haoyu Tang and Rui Jin performed the bioinformatic analyses. Weina Lu, Miao Li, and Xinwei Geng wrote the first draft of the article. Yanping Wu, Chen Zhu, and Min Zhang reviewed and edited the manuscript.

## CONFLICT OF INTEREST STATEMENT

The authors declare no conflicts of interest related to this work.

## FUNDING

This research was supported by the National Natural Science Foundation of China (82270023, U22A20265, 82225001, 81920108001, 82300102), and the Development Project of Zhe‐jiang Province's “Lingyan” (No. 2023C03067).

## ETHICS STATEMENT

All animal procedures were conducted in accordance with institutional and national guidelines for laboratory animal care, with prior approval from the Animal Ethics Committee of Zhejiang University (Approval No. ZJU20200159). For human sample studies, formalin‐fixed, paraffin‐embedded tissues obtained from surgical resections or biopsies were retrieved from the archives of the Department of Pathology, Second Affiliated Hospital of Zhejiang University School of Medicine. The use of human tissue samples was reviewed and approved by the Human Research Ethics Committee of the same institution, with a waiver of informed consent granted (Approval No. 2025‐0526).

## Supporting information



Supporting Information

Supporting Information

## Data Availability

Data utilised in this study were retrieved from publicly accessible platforms, including the Kaplan–Meier Plotter (https://www.kmplot.com/), UCSC Xena Browser (https://xena.ucsc.edu/), and UALCAN (https://ualcan.path.uab.edu/). Immunohistochemical profiles of PICH in lung cancer were accessed via The Human Protein Atlas (https://www.proteinatlas.org/). Materials and data from this study are available upon reasonable request from the corresponding authors.
