# Supplementary material for: PICH deficiency attenuates the progression of lung adenocarcinoma and disrupts the DNA damage response
Source: Clin Transl Med. 2025 May 29;15(5):e70349. doi: 10.1002/ctm2.70349 (PMC12122185; doi:10.1002/ctm2.70349)
Supplement: Supplementary file 1 — Supporting Information [file CTM2-15-e70349-s002.docx]

**SUPPLEMENTARY DATA**


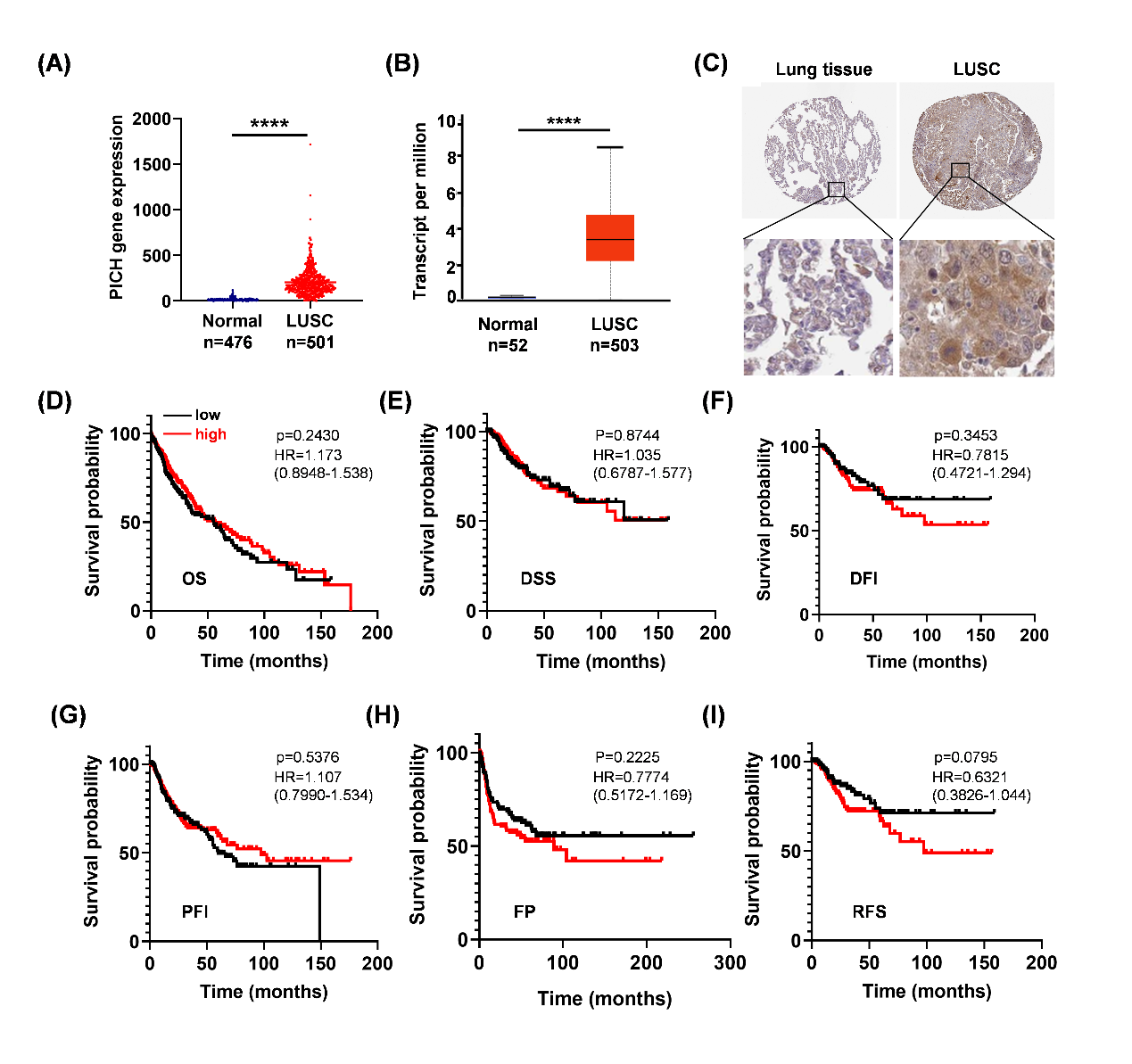


**FIGURE S1. Relevance of PICH expression in lung squamous carcinoma and its prognostic implications for patients.**

(A) PICH mRNA levels were analyzed in normal lung and LUSC tissues retrieved from the Kaplan Meier plotter database. (B) UALCAN was employed to investigate PICH expression in normal lung and LUSC tissues. (C) Immunohistochemical staining of PICH was performed on normal lung and LUSC tissues obtained from The Human Protein Atlas database. (D) Overall survival analysis of LUSC patients involved stratification based on high or low expression levels of PICH. (high: n=269; low: n=225). (E) Disease specific survival analysis in LUSC patients (high: n=238; low: n=204). (F) Disease free interval analysis in LUSC patients (high: n=163; low: n=137). (G) Progression free interval analysis in LUSC patients (high: n=270; low: n=225). (H) First progression analysis in LUSC patients (high: n=110; low: n=110). (I) Relapse free survival analysis in LUSC patients (high: n=150; low: n=150). The data were presented as means ± SEM. Student's t-test analysis was used in A, B plots, log-rank test analysis was utilized in D-I plots, ****p < 0.0001.


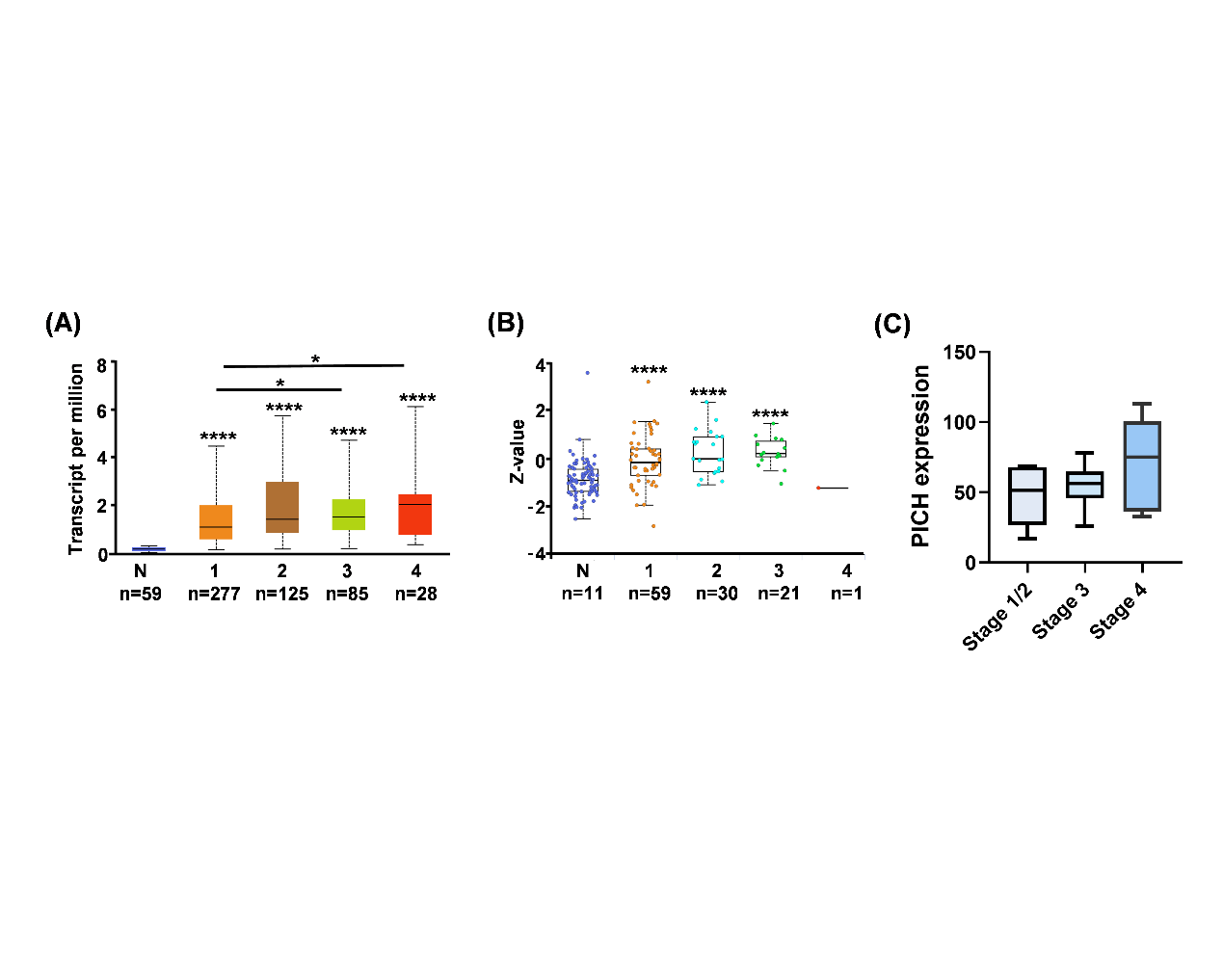


**Figure S2. PICH expression level was positively correlated with LUAD stage.**

(A) UALCAN was employed to analyze the mRNA levels of PICH at different stages of LUAD. (B) CPTAC data were used to investigate PICH protein levels across various stages of LUAD. (C) PICH expression at different stages of LUAD assessed by immunohistochemical staining (stage1/2, n = 8; stage 3, n =6; stage 4, n =6). One-way ANOVA was used in A and B plots, ****p < 0.0001, *p < 0.05.


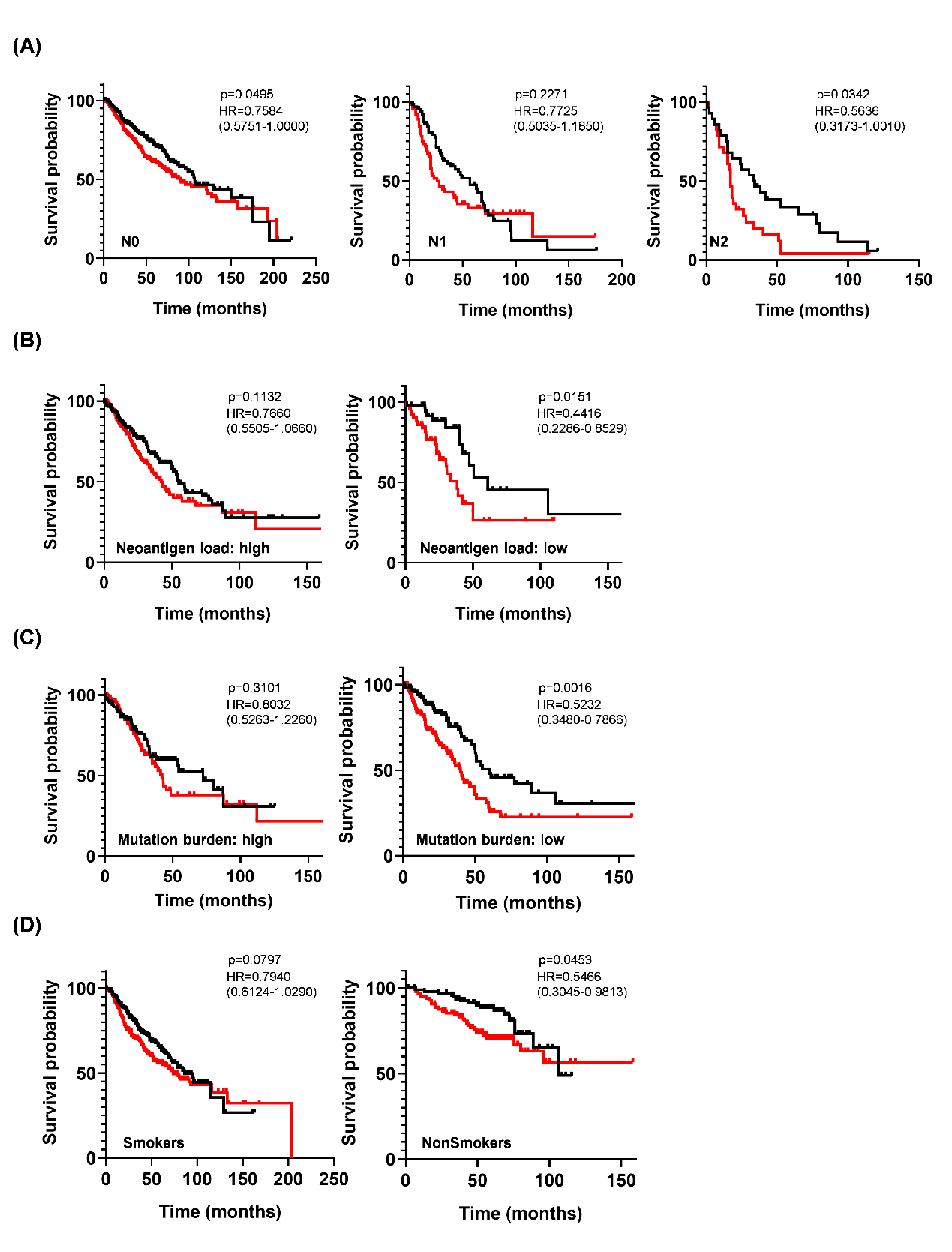


**FIGURE S3. Stratified survival analysis evaluating the association between PICH expression and prognosis in LUAD patients.**

(A) Kaplan–Meier curves showing overall survival (OS) in LUAD patients stratified by PICH expression across lymph node metastasis subgroups: N0 (high, n = 240; low, n = 241), N1 (high, n = 65; low, n = 65), and N2 (high, n = 28; low, n = 28). (B) OS comparison based on PICH expression in patients with high versus low neoantigen load (high neoantigen group high n=191; low n=191; low neoantigen group high n=52; low n=51). (C) OS in patients stratified by PICH expression and tumor mutation burden: high mutation group (high n = 125; low n = 126), and low mutation group (high n = 121; low n = 123). (D) OS curves for smokers and non-smokers according to PICH expression (smoker group: high n = 270, low n = 274; non-smoker group: high n = 96, low n = 96). Log-rank test analysis was utilized in A-D plots.
