# Supplementary material for: PICH deficiency attenuates the progression of lung adenocarcinoma and disrupts the DNA damage response
Source: Clin Transl Med. 2025 May 29;15(5):e70349. doi: 10.1002/ctm2.70349 (PMC12122185; doi:10.1002/ctm2.70349)
Supplement: Supplementary file 2 — Supporting Information [file CTM2-15-e70349-s001.docx]

**MATERIALS AND METHODS**

**1 Cell culture and transfection**

Human cell lines 293T, A549, and H1299 (all sourced from ATCC) were maintained in DMEM containing 10% fetal bovine serum (Gibco) and incubated at 37°C in a humidified atmosphere with 5% CO₂. To achieve stable knockdown of PICH, shRNA constructs were inserted into the pLVX-U6-ZsGreen vector and transduced into A549 and H1299 cell lines (RRID: CVCL_0023 and CVCL_0060, respectively). The sequences used for PICH-targeting shRNAs were: shRNA-1, CCTGAAGATTATCCAGAAGAA; and shRNA-2, GCAGCTTCAGTCTTTGCATGC. Lentiviral packaging was achieved by co-transfecting the plasmids with psPAX2 and pMD2.G into 293T cells, and the transfection was maintained for 48 hours. Viral particles were harvested from the culture supernatant, concentrated by ultrafiltration, and then applied to infect A549 and H1299 cells.

**2 Antibodies and agents**

For western blot analysis, the following primary antibodies were used: rabbit anti-PICH (Cell Signaling Technology, #8886, RRID: AB_11128848), mouse anti-β-ACTIN (Proteintech, #66009-1, RRID: AB_2687938), rabbit anti-Cleaved Caspase-3 (Cell Signaling Technology, #9661, RRID: AB_2341188), rabbit anti-Cleaved Caspase-7 (Cell Signaling Technology, #8438, RRID: AB_11178377), rabbit anti-Cleaved Caspase-9 (Cell Signaling Technology, #9505, RRID: AB_2290727), rabbit anti-PUMA (Cell Signaling Technology, #4976, RRID: AB_2064551), rabbit anti-Cleaved PARP (Cell Signaling Technology, #5625, RRID: AB_10699459), mouse anti-P53 (Santa Cruz, #sc-126, RRID: AB_628082). The secondary antibody used was HRP-conjugated goat anti-mouse IgG (Huabio, #HA1006).

For immunohistochemistry, mouse tissue sections were stained with: rabbit anti-PICH (Cell Signaling Technology, #8886, RRID: AB_11128848), rabbit anti-γH2AX (Abcam, #ab81299, RRID: AB_1640564), rabbit anti-Ki67 (Abcam, #ab15580, RRID: AB_443209), rabbit anti-Cleaved Caspase-3 (Cell Signaling Technology, #9661, RRID: AB_2341188). Human lung samples were stained with rabbit anti-PICH (Sigma-Aldrich, #HPA050492, RRID: AB_2681145).

For immunofluorescence experiments, the following antibodies were used: mouse anti-γH2AX (Millipore, #05-636, RRID: AB_309864), Alexa Fluor™ 555-conjugated goat anti-mouse IgG (H+L), cross-adsorbed (Invitrogen, #A-21422).

**3 Bioinformatic analysis**

Bioinformatics analyses were conducted using several online platforms, including Kaplan–Meier Plotter (https://www.kmplot.com/), UCSC Xena (https://xena.ucsc.edu/), and UALCAN (https://ualcan.path.uab.edu/). Immunohistochemical data for PICH expression in lung cancer were retrieved from The Human Protein Atlas (https://www.proteinatlas.org/).

**4 Clinical specimens**

To investigate PICH expression in human lung adenocarcinoma, paraffin-embedded tissues from 30 patients who underwent surgical resections or biopsies were retrieved from the archives of the Department of Pathology, Second Affiliated Hospital, Zhejiang University School of Medicine. Immunohistochemical evaluation of PICH expression was conducted across three clinical subsets:(1) tumor samples and paired adjacent non-tumorous tissues (n = 25); (2) patients categorized by survival outcome within three years post-diagnosis (survivors: n = 23; deceased: n = 7); (3) different tumor stages based on the TNM staging system (stage 1/2: n = 8; stage 3: n = 6; stage 4: n = 6). The study protocol received approval from the Human Research Ethics Committee of the institution (Approval No. 2025-0526), with informed consent waived.

**5 Immunohistochemical**

Human and mouse tissues were fixed in 4% paraformaldehyde overnight and embedded in paraffin. Tissue sections (5 μm thick) were prepared using LEICA RM2235 microtome. Following deparaffinization and rehydration, endogenous peroxidase activity was quenched with 3% hydrogen peroxide. Antigen retrieval was performed by macrowaving the slides in sodium citrate buffer for 20 minutes. Following a cooling step, to minimize nonspecific binding, sections were blocked with 5% BSA in PBS for 30 minutes at room temperature. Primary antibody incubation was then carried out overnight at 4 °C, followed by a 20-minute incubation with secondary antibodies (ZSGB-BIO) at 37 °C. Color development was achieved using DAB (ZSGB-BIO, ZLI-9018), and re-dying with hematoxylin. Stained sections were imaged using an Olympus VS200 digital slicing scanner.

Immunohistochemical signal evaluation was conducted using both semi-quantitative and software-assisted approaches. In human samples, PICH expression was assessed using the H-score system, calculated as: H-score = [0 × (% of negative cells)] + [1 × (% weak positive)] + [2 × (% moderate positive)] + [3 × (% strong positive)]. For murine lung tissue, Ki67-positive cell proportions were quantified using QuPath software, γH2AX and cleaved caspase 3 expression levels were measured via Image J.

**6 Western blotting**

Total cellular proteins were isolated on ice using RIPA buffer (Beyotime) supplemented with protease and phosphatase inhibitors (Roche). Protein levels were quantified using the BCA protein assay kit (ThermoFisher). Equal amounts of lysates were added with 5× SDS loading buffer and denatured by boiling at 100 °C for 10 minutes. Proteins were separated by SDS-PAGE with gel concentrations (6%, 10%, or 10–20% gradient) selected based on molecular weight and subsequently transferred to PVDF membranes (Millipore) using a wet transfer system. After blocking with 5% non-fat milk in TBST for 1 hour at room temperature, membranes were incubated overnight at 4 °C with the appropriate primary antibodies. HRP-conjugated secondary antibodies were applied for one hour at ambient temperature. Signal bands were imaged using a Bio-Rad ChemiDoc system.

**7 Clone formation assay**

After 48 hours of shRNA transfection, cells were reseeded into 6-well plates at a density of 500 cells per well. Clonal expansion was monitored over a 14-day period until colonies became visible. Cells were fixed in 4% paraformaldehyde for 30 minutes at room temperature, followed by staining with 0.5% crystal violet for 1 hour. The plates were then gently rinsed with distilled water, allowed to air-dry completely, and subsequently imaged. The number of visible colonies was quantified using the Image J software.

**8 Apoptosis analysis**

Cells were harvested by centrifugation at 4 °C, followed by three times wash with PBS. Subsequently, the samples were labeled with Annexin V (MultiSciences, 70-APCC101-100) dye for 15 minutes at room temperature. The fluorescent signal was then analyzed on a Beckman flow cytometer using the CytExpert software.

**9 Comet assay**

The cells were digested with 0.25% trypsin containing EDTA, resuspended in ice-cold PBS at a density of 1×10^6^/mL. Adhesive slides were pre-warmed at 37 ℃, and three layers of agarose gel were sequentially added and solidified at 4 ℃. The base layer consisted of 100 µL of 0.5% dissolved normal melting point agarose (NMA). The middle layer was composed of a mixture containing 10 µL of cell suspension and 75 µL of low melting point agarose (LMA) at a concentration of 0.7%. The top layer comprised 100 µL of 0.7% LMA. Subsequently, the slides were immersed in lysis buffer (KeyGEN) at 4 ℃ for 1.5 hours, followed by two times wash with PBS. To unwind and denature the DNA, an alkali solution (1 mM EDTA, 300 mM NaOH) was utilized prior to electrophoresis at 25V for 25 minutes. Following electrophoresis, the slides were neutralized with 0.4 mM Tris-HCl (pH 7.5) three times at 4℃, followed by PI staining. Images were captured using an automated Nikon Eclipse Ni microscope. Olive tail moments were quantified using Casplab software.

**10 Immunofluorescence**

Cells were fixed in freshly prepared 4% formaldehyde for 15 minutes at room temperature, followed by 20 minutes of permeabilization with 0.5% Triton X-100 to enhance antibody penetration. To reduce nonspecific binding, cells were blocked with 3% bovine serum albumin (BSA) in PBS for a minimum of 1 hour. Primary antibodies were subsequently applied and incubated overnight at 4 °C. After extensive washing with PBS, cells were incubated for 30 minutes at room temperature with species-appropriate secondary antibodies conjugated to fluorophores. Nuclei were counterstained using DAPI. Fluorescence signals were visualized and recorded using a Nikon Eclipse Ni imaging system, and images were analyzed via NIS-Elements software (Nikon).

**11 Mouse genotyping**

*Pich^flox/flox^* mice were generously provided by Dr. Andres J Lopez-Contreras, University of Copenhagen. *Kras^G12D^* mice were kindly provided by Prof. Hongbin Ji, Center for Excellence in Molecular Cell Science, CAS. The genotyping primers used were as follows:

*Pich*^flox/flox^

*Pich*-F1 CTATGCCTGATCCTCCCCAG

*Pich*-R1 GCTAACAGACAAAATGGCCCT

*Pich*-F2 AAAGCCCAACTACAGTGTGG

*Kras*^G12D^

*Kras*-F CTAGCCACCATGGCTTGAGT

*Kras*-R TCCGAATTCAGTGACTACAGATG

**12 Genetically engineered mouse model**

*Kras^G12D^* and *Kras^G12D^*-*Pich^flox/flox^* mice aged 6 to 8 weeks were anesthetized via intraperitoneal injection of 0.1 mL 1% pentobarbital sodium prior to intratracheal administration of 5 × 10⁷ plaque-forming units (PFU) of Ad-Cre virus. Mice were monitored post-infection and sacrificed at 20 to 24 weeks for downstream analysis. For survival analysis, humane endpoints were defined as the appearance of clinical signs such as rapid or labored respiration, decreased food intake or mobility, significant lethargy, or a body weight reduction exceeding 20% of the baseline measurement. All animal-related procedures complied with institutional and national ethical standards and were conducted under approval from the Zhejiang University Institutional Animal Care and Use Committee (Protocol No. ZJU20200159).

**13 Hematoxylin-eosin staining**

Lung tissues were collected and fixed overnight in 4% formaldehyde, then dehydrated through graded ethanol solutions and embedded in paraffin. Paraffin blocks were sectioned into 5 μm slices using a LEICA RM2235 microtome. Sections were dewaxed in xylene and rehydrated sequentially in descending ethanol concentrations. Hematoxylin and eosin (H&E) staining was performed to assess tumor burden. The proportion of tumor area relative to total lung area was calculated based on scanned slide images. Quantification was carried out using the Image J software.

**14 Statistical Analysis**

Statistical analysis was performed using GraphPad Prism 8.0 software. Continuous variables were expressed as mean ± standard error of the mean (SEM). For two-group comparisons, a two-tailed Student’s t-test was employed, while differences among multiple groups were assessed using one-way ANOVA followed by Dunnett’s multiple comparisons test. Kaplan–Meier survival analysis was conducted, with group differences evaluated using the log-rank test. Statistical significance was defined as P < 0.05 for all analyses.
